# Upregulation of miRNA-200c during Disease Progression in COVID-19 Patients

**DOI:** 10.3390/jcm12010283

**Published:** 2022-12-29

**Authors:** Lukas van de Sand, Peer Braß, Jonas Gregorius, Kevin Pattberg, Andrea Engler, Ulf Dittmer, Christian Taube, Stephan Brock, Marc Moritz Berger, Thorsten Brenner, Oliver Witzke, Adalbert Krawczyk

**Affiliations:** 1 Department of Infectious Diseases, West German Centre of Infectious Diseases, University Duisburg-Essen, 45147 Essen, Germany; 2 Department of Anesthesiology and Intensive Care Medicine, University Hospital Essen, University Duisburg-Essen, 45147 Essen, Germany; 3 Institute for Virology, University Hospital Essen, University of Duisburg-Essen, 45147 Essen, Germany; 4 Department of Pneumology, University Medicine Essen-Ruhrlandklinik, University Duisburg-Essen, 45147 Essen, Germany; 5 Molecular Health GmbH, 69115 Heidelberg, Germany

**Keywords:** SARS-CoV-2, COVID-19, miRNA-200c

## Abstract

The COVID-19 pandemic has caused more than 6 million deaths worldwide since its first outbreak in December 2019 and continues to be a major health problem. Several studies have established that the infection by SARS-CoV-2 can be categorized in a viremic, acute and recovery or severe phase. Hyperinflammation during the acute pneumonia phase is a major cause of severe disease progression and death. Treatment of COVID-19 with directly acting antivirals is limited within a narrow window of time between first clinical symptoms and the hyperinflammatory response. Therefore, early initiation of treatment is crucial to assure optimal health care for patients. Molecular diagnostic biomarkers represent a potent tool to predict the course of disease and thus to assess the optimal treatment regimen and time point. Here, we investigated miRNA-200c as a potential marker for the prediction of the severity of COVID-19 to preventively initiate and personalize therapeutic interventions in the future. We found that miRNA-200c correlates with the severity of disease. With retrospective analysis, however, there is no correlation with prognosis at the time of hospitalization. Our study provides the basis for further evaluation of miRNA-200c as a predictive biomarker for the progress of COVID-19.

## 1. Introduction

Severe acute respiratory syndrome coronavirus 2 (SARS-CoV-2), causing coronavirus disease 2019 (COVID-19), represents a global public health emergency. Since COVID-19 was declared a pandemic by the world health organization (WHO) on 12 March 2020, the virus has caused 500 million human infection cases worldwide, killing more than 6.4 million people (to date) worldwide [1]. With a high mutation rate, SARS-CoV-2 evolved rapid resistance to current antiviral drugs and vaccines [2]. Different variants of SARS-CoV-2 have been identified so far. The delta variant, known for its severe disease course and increased transmissibility, was designated as a variant of concern until November 2021 [2]. Currently, omicron is the predominant variant causing the fourth wave of COVID-19 to sweep the globe [3]. Severity and infectivity differ between the variants. Regardless of the variant and vaccination status, those patients with comorbidities, such as immunosuppression, hypertension, diabetes, obesity and others, are more likely to have severe disease and ongoing complications associated with COVID-19 [4]. Infected patients have initially minor common cold symptoms, which in severe cases may rapidly evolve to severe respiratory failure. Acute respiratory distress syndrome (ARDS) is present in 53–95% of critical cases in intensive care units (ICUs) [5]. In about 86% of all COVID-19-related deaths, ARDS is responsible for the fatality [5]. The analysis of post-mortem COVID-19 lung tissue suggests two distinct stages of disease progression [6]. Early in the disease, a high viral load and high expression of cytokines and interferon-inducible genes (ISGs) and sparse immune infiltrates have been identified, while late in the disease, a low viral load coincides with low local expression of cytokines and ISGs, and strong infiltration of macrophages and lymphocytes [6]. Patients who die in the early phase are not able to adequately control SARS-CoV-2, while patients who die in the late phase suffer from diffuse tissue damage and immunopathology [6]. Seemingly, in the late disease stage, pathogenesis is decoupled from the acute viral load. Notably, different molecular factors may be decisive for therapeutic success and prognosis.

MicroRNAs (miRNAs) are small non-coding RNA molecules that can negatively regulate gene expression. In most cases, they target messenger RNA (mRNA) by interacting with the 3′-untranslated region (3′-UTR) [7]. This mechanism controls at least 30% of all protein-coding regions [8]. Furthermore, miRNAs regulate several other regions, such as the 5′ UTR, coding sequence and gene promoters [9]. Several studies have reported that miRNAs play an important role in the prognosis, diagnosis and evaluation of the therapeutic response in many human cancers, cardiac hypertrophy and failure, inflammation and infectious diseases [7]. One of these significantly enriched miRNAs is miRNA-200c, a negative regulator of angiotensin-converting enzyme 2 (ACE2) expression [10]. ACE2 is known to be a specific functional entry receptor for SARS-CoV-2 [11]. Physiologically, ACE2 is a membrane-bound enzyme that inactivates angiotensin II (Ang II) by cleaving it to produce Ang 1–7 [12]. Dysregulation of ACE2 counteracts with the development of ARDS according to the predominant pro-inflammatory effect of Ang II and suppression of the antiinflammatory effects of Ang 1–7. In addition, Ang II mediates numerous effects such as blood pressure regulation, electrolyte regulation, cell proliferation, fluid balance, fibrosis and hypertrophy [13]. Downregulation of ACE2 expression is likely to increase the extent of inflammation in ARDS induced by viral infections [14]. ACE2 is expressed predominantly in epithelial cells of the intestine and respiratory tract [15]. In this sense, out of many different possible ACE2 regulator miRNAs, miRNA-200c is the most promising. miRNA-200c is induced by oxidative stress and miRNA-200c levels are highly elevated in plasma from pneumonia patients, positively correlated with severity of interstitial lung disease and elevated in COPD patients [16,17,18,19]. SARS-CoV-induced ARDS is associated with the downregulation of ACE2, which is mediated through an increase in miRNA-200c [20].

Recently, it has been shown that upregulated circulating miRNA-200c in plasma may increase the risk of obese individuals to severe COVID-19 [8]. On the other hand, miRNA-200c is a biomarker of insulin resistance in obesity [21]. miRNA-200c diminishes insulin production by inducing pancreatic β-cell damage, while its suppression improves β-cell function in Type 2 diabetes and restores endothelial function [22,23]. miRNA-200c downregulates IRS1, which is associated with insulin resistance [24,25]. In summary, miRNA-200c may play an important role in molecular pathologies associated with disease severity, but its potential as a prediction marker for ICU treatment or fatal outcome remains unclear.

## 2. Materials and Methods

### 2.1. Study Design

In the present study, we investigated miRNA-200c as a potential marker for predicting the severity of COVID-19. All participants were hospitalized at the University Hospital Essen with a worst WHO score of at least four. In total, the study included 113 patients from whom samples were collected at two time points (onset (time of hospitalization): *n* = 111 and day 7: *n* = 37). The group consisted of 39 women and 72 men and the median age was 64 years. The average BMI of the subjects was 27.8 (interquartile range 24.6–33.3). Participants suffered from myocardial infarction (*n* = 13), chronic lung disease (*n* = 18) and diabetes (*n* = 18) before being hospitalized for COVID-19. During hospitalization, a total of 19 patients died. The paired sample group consisted of patients from whom blood samples were available at both time points (*n* = 35). Table 1 shows detailed information. All Helsinki principles were considered in the present study and approved by the local ethics committee of the University Hospital Essen, Germany (21-10026-BO). All probands consented to participation in the study.

### 2.2. Sampling

**Figure 1:**
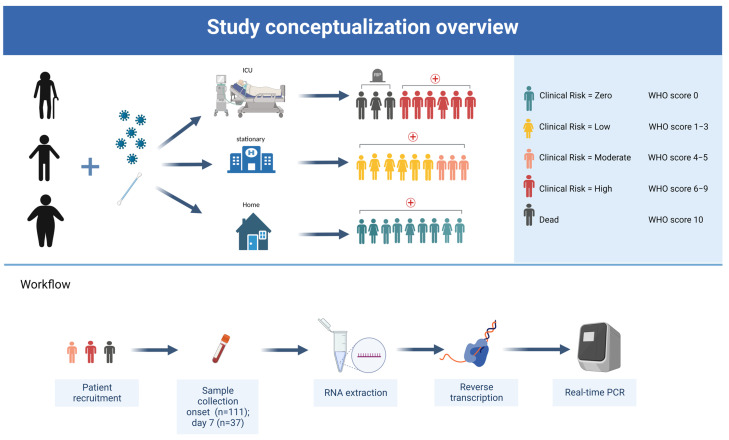
Study conceptualization overview. COVID-19 positive tested, hospitalized patients were included in the study irrespective of their age and pre-existing conditions. The clinical progression grade scale provides a measure of illness severity from 0 (not infected) to 10 (dead). Patients assigned to the low-risk group are either asymptomatic (WHO 1) or have mild symptoms that can be treated at home (WHO 2–3). Hospitalized patients at standard care ward can be categorized as those who do not need oxygen therapy (WHO 4) and those who need oxygen assistance by mask or nasal prongs (WHO 5). Severe disease cases with high flow oxygen therapy or mechanical ventilation are assigned WHO scores 6 to 9. The study included a patient population with a retrospective WHO score of at least four. miRNA sample collection was performed at hospitalization (onset) and seven days later (day 7).

Severely ill COVID-19 patients over 18 years of age, without a reported prior SARS-CoV-2 infection, were recruited between April 2020 and April 2022 after a PCR-confirmed SARS-CoV-2 infection (Figure 1). The patients were hospitalized at the University Hospital in Essen, Germany, and treated at the intensive care unit (ICU) or standard ward. Initially, 111 patients were included in our study; for our further analysis we only selected those with a sample collection at two time points (onset and day 7). One subject was vaccinated with the mRNA-based SARS-CoV-2 vaccine Comirnaty and another with Janssen COVID-19 vaccine. After informed consent from all participants was obtained, blood samples (7.5 mL S-Monovette^®^ EDTA Gel, Sarstedt AG, Nümbrecht, Germany) for measurement of miRNA-200c levels were collected at the first day of hospitalization, as well as on the seventh day. Plasma collection was performed by sample centrifugation at 3500× *g* rounds per minute for 30 min at room temperature. Clinical data from same-day routine laboratory measurements of hospitalized COVID-19 patients were collected according to local standards (potassium EDTA for determination of leukocytes and hemoglobin, serum gel for determination of C-reactive protein (CRP) and lactate dehydrogenase (LDH); Sarstedt AG, Nümbrecht, Germany). For automated measurement of these laboratory parameters, the following devices were used: ADVIA1 1800 Clinical Chemistry System (Siemens Healthcare Diagnostic, Erlangen, Germany) for CRP and LDH and XN-1000 Pure (Sysmex, Norderstedt, Germany) for hematological blood count.

### 2.3. Quantitative miRNA-200c Real-Time PCR

Following the standard protocol, miRNA was extracted in RNase-free environment using miRNeasy Serum/Plasma Kit (Qiagen, Hilden, Germany) according to the manufacturer’s instructions. For miRNA expression analysis, cDNA was generated by stem-looped miRNA-specific reverse transcription primers for miRNA-16 (Lot No. P220107-002F10) and miRNA-200c (Lot No. P220107-002F09) by TaqMan™ MicroRNA Assay (Assay-IDs: 000391, 002300; Thermo Fisher Scientific, Cleveland, OH, USA). The cDNA synthesis was carried out from 2 µg miRNA per sample following the TaqMan™ Small RNA Assay protocol. The reactions were incubated at 16 °C for 30 min, 42 °C for 30 min and 85 °C for 5 min. The quantitative real-time PCR (qRT-PCR) was performed according to the TaqMan™ MicroRNA Assay protocol. Target sequences were expanded in a 20 μL reaction mixture containing 10 μL of TaqMan™ Universal Master Mix II (Thermo Fisher Scientific), 1 μL of 20× TaqMan miRNA primer for miRNA-200c or miRNA-16, 1.33 μL of cDNA and 7.67 μL of DNase-free water. The conditions of the PCR cycles were 10 min at 95 °C followed by 40 cycles of 15 s at 95 °C and 1 min at 60 °C. Amplified PCR products were detected by StepOne™ Real-Time PCR System (Thermo Fisher Scientific). The relative quantification (RQ) was calculated by the 2^−ΔΔCt^ method and miRNA expression levels were normalized to housekeeping gene region via miRNA-16 and healthy control plasma [26].

### 2.4. Statistical Analysis

All statistical analyses were performed using GraphPad Prism version 9 (GraphPad Software Inc., La Jolla, CA, USA). All experiments were performed in triplicate. Data are expressed as the geometric mean ± standard deviation and an unpaired 2-tailed *t*-test was performed to calculate the significance between the groups. The results were considered significant when *p* ≤ 0.05.

## 3. Results

In the present study, we investigated miRNA-200c expression in severe COVID-19 patients after hospitalization by qRT-PCR. Furthermore, we analyzed if there is a correlation between miRNA-200c levels and serum lactate dehydrogenase levels, which is known to be a prognostic factor for disease progression and prognosis in critically ill patients.

### 3.1. Correlation between miRNA-200c Levels and the Severity of COVID-19

**Figure 2:**
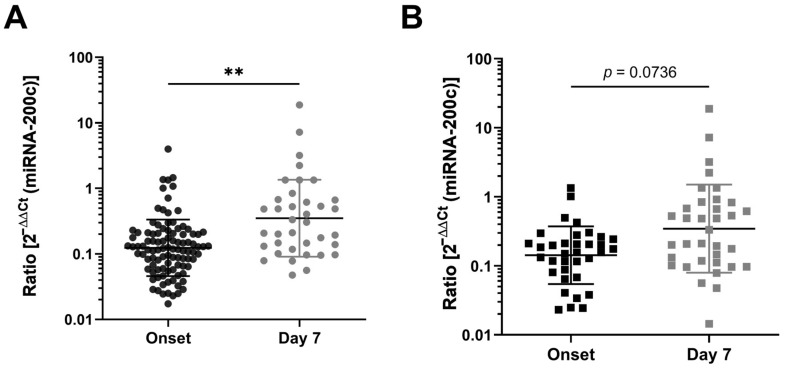

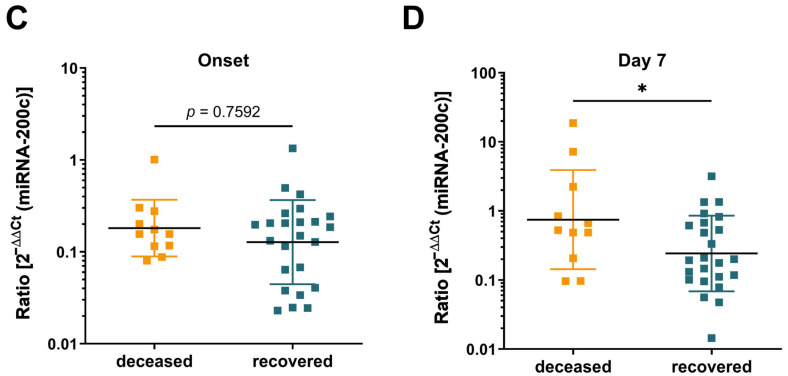
miRNA-200c expression levels in critically ill COVID-19 patients. (**A**) Quantitative RT-PCR analysis of miRNA-200c expression of all patients at the time of hospitalization (onset) and at day seven after hospitalization. (**B**) miRNA expression of patients with study enrollment at both time points (paired sample group; *n* = 35). (**C**) Expression levels of miRNA-200c at study enrollment (onset) of the paired sample group according to the clinical outcome of disease. (**D**) Expression of miRNA-200c in paired sample group at day 7 according to the clinical outcome of disease. The miRNA-200c levels were measured in triplicate. Each dot represents the median value from three measurements. The ratio represents the miRNA-200c values normalized to a healthy control group and the housekeeping gene miRNA-16. The mean values of relative quantification (ratio) of plasma miRNA-200c levels are indicated by horizontal lines. Error bars indicate the standard deviation of the mean. *p*-values were determined by an unpaired *t*-test. * *p* < 0.05, ** *p* < 0.01.

To evaluate miRNA-200c as a predictive marker, we collected plasma from patients at the time of hospitalization (onset) and during their hospital stay at day 7, quantified the miRNA-200c plasma levels and compared them with the clinical outcome of the patients. In total, we investigated samples from 111 patients at onset and 37 samples at day seven upon onset. The overall cohort showed a significant increase in the miRNA-200c expression levels between onset and day 7 (*p* = 0.004) when analyzing the overall samples. On average, the expression level increased 6.9-fold (Figure 2A). Of these, 35 patient samples were paired (onset and day 7). There was also an evident increase in the miRNA-200c levels on day seven when considering those patients with samples from onset and day seven (*n* = 35) but this did not reach the level of significance (*p* = 0.075). On average, the paired samples showed an approximately 5.6-fold increase in the miRNA-200c expression (Figure 2B). No significant differences were detected between the miRNA-200c levels at onset in patients who deceased from the infection and those who recovered (Figure 2C; *p* = 0.759). Interestingly, patients who deceased after seven days of intensive care treatment had on average 5.8-fold higher miRNA-200c levels than patients who recovered from COVID-19 (Figure 2D; *p* = 0.047).

### 3.2. Correlation between LDH Levels and the Severity of COVID-19

**Figure 3:**
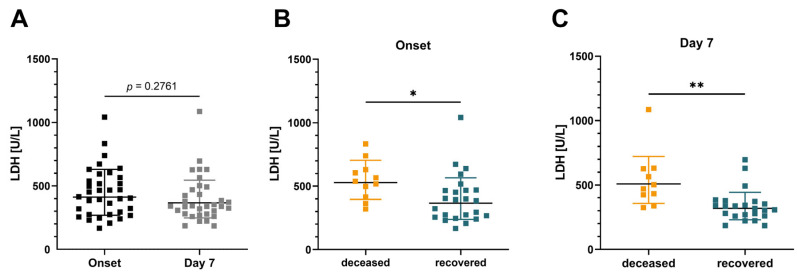
Correlation between LDH levels and the clinical outcome of infection. (**A**) Analysis of LDH values of the paired sample group at onset and day 7. Correlation between the LDH levels and the clinical outcome of infection when assessed at onset (**B**) or day 7 (**C**) of hospitalization. Graphs show the geometric mean and standard deviation. *p*-values were determined by an unpaired *t*-test. * *p* < 0.05, ** *p* < 0.01.

Clinical markers of infection such as LDH were routinely determined to assess the patient status. According to the used assay, the refencing range of LDH is 135 to 225 U/L in adults. The measured LDH values for the paired sample group showed no significant difference between onset and day seven (Figure 3A; *p* = 0.276). As expected, at the individual time points, there were differences in the LDH levels between deceased and recovered patients at both onset (*p* = 0.037) and day seven (*p* = 0.002). LDH was elevated in patients who deceased due to COVID-19 (Figure 3B,C).

### 3.3. Correlation between miRNA-200c and Lactate Dehydrogenase

**Figure 4:**
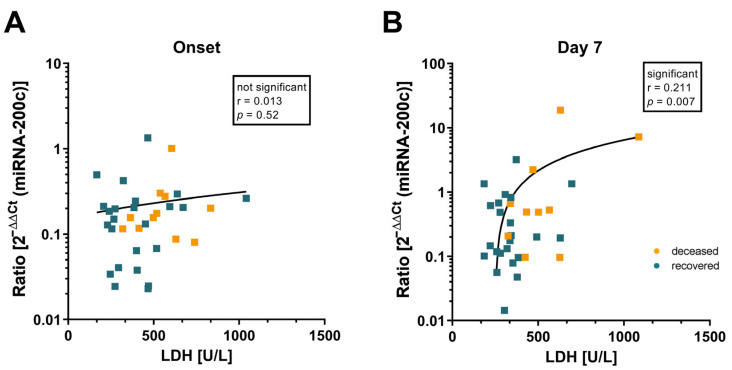
Correlation between miRNA-200c and LDH. Scatter plots of Spearman correlation analysis show the correlation between miRNA-200c and LDH at onset (**A**) and day 7 (**B**). Color-coded values indicate a fatal or non-fatal course of infection for the patient.

Next, we investigated if there is a correlation between miRNA-200c and LDH levels. Both infection markers might be good candidates to be further evaluated as potential markers to predict the course of disease. We analyzed the correlation between these markers for the paired samples (onset and day 7) (Figure 4). Interestingly, although both parameters did not correlate at onset (Figure 4A), there was a positive correlation between LDH and miRNA-200c levels at day seven (*p* = 0.007; Figure 4B).

## 4. Discussion

In the present study, we analyzed whether miRNA-200c is a suitable marker to predict the course of COVID-19. Diagnostic predictive tools are urgently needed to improve the treatment of COVID-19 patients. Such a reliable prediction of the course of disease may help to quickly adjust the optimal antiviral or immunomodulatory treatment of the patient and, thus, to improve the clinical outcome. Various miRNAs have already been shown to have regulatory effects and to correlate with the severity of infection in other viral diseases [27,28]. Here, we investigated if miRNA-200c correlates with the severity of COVID-19 and thus may serve as a prediction marker to forecast the course of disease.

The SARS-CoV-2 virus was previously discovered to use a pulmonary angiotensin-converting enzyme (ACE2) as a receptor for entering pulmonary cells [11]. An increased miRNA-200c expression leads to downregulation of ACE2 [17]. Accordingly, a prior study indicated that enhanced miRNA-200c levels may correlate with a milder course of disease [29]. However, in contrast to this hypothesis, it has been shown that miRNA-200c expression may be associated with a worsening clinical outcome [30].

Our study cohort consisted of severely ill COVID-19 patients treated at the University Hospital. Overall, we found that there is a correlation between the miRNA-200c levels and the outcome of disease. Enhanced miRNA-200c levels correlated with a lethal outcome of infection. When looking at the paired samples from onset and day seven obtained from 35 patients, elevated miRNA-200c levels also correlated with a lethal outcome of infection but did not reach the level of significance (*p* = 0.075). Interestingly, when analyzing the miRNA-200c values at onset, there was no correlation between the miRNA-200c levels and the clinical outcome of disease. Interestingly, miRNA-200c levels increased with the progression of the disease and were elevated in samples taken at day seven, resulting in a statistically significant increase in miRNA-200c in patients who deceased compared to patients that recovered.

Similar findings were reported for other infectious diseases. For instance, miRNA-200c expression levels also progressively increase during the course of H5N1 virus infection [17]. The influenza virus induces the upregulation via non-structural protein 1 (NS1) and the accommodation of viral RNA in the cells [17]. The latter could lead to increased expression of miRNA-200c via activation of the NF-κB pathway [31]. Similarly, upregulation of miRNA-200c in bacterial inflammation is mainly mediated by the NF-κB pathway, which is activated by bacterial lipopolysaccharides (LPS) [32,33]. However, it needs further investigation as to whether miRNA-200c upregulation in severely ill COVID-19 patients may also lead to increased NF-κB activation and inflammation, which is associated with a more severe course of disease. Upregulation of miRNA-200c expression levels was also observed in hepatitis B- and hepatitis C-infected patients [17]. Thus, miRNA-200c seems to play an important role in many different infectious diseases. Notably, downregulation of ACE2 appears to play a particularly important role in ARDS, relevant to both viral and bacterial lung infections. The influenza virus also leads to ARDS in severe courses, as does SARS-CoV-2 in our study. This supports the hypothesis that strong expression of miRNA-200c is commonly observed in severe and lethal courses of COVID-19. However, the exact molecular mechanisms regulating the increase in miRNA-200c levels and its impact on the respective disease remain unknown. One possible explanation might be the virus-induced kallikrein–kinin system (KKS) pathway activation [34]. Activation of KKS results in the release of bradykinin 1 receptor (B1R)-positive endothelial microvesicles (MVs). Thus, B1R signaling induces the excretion of B1R bearing microvesicles in an auto-amplifying manner [35]. Such MV-based intercellular communication also occurs between different airway cells [10]. In addition to proteins, the secretome and exosomes secreted by airway epithelial cells also contain miRNAs [36]. Interestingly, one of these significantly enriched miRNAs in B1R-positive MVs is miR200c [8,35]. As a result, a possible pathogenic mechanism of miRNA-200c upregulation may be that targeting antioxidant proteins leads to an increase in reactive oxygen species (ROS) formation [16,37], which contributes to the progression of the inflammatory process and endothelial barrier dysfunction [38]. In addition, miRNA-200c has been shown to directly target forkhead box O1 (FOXO1) [37]. FOXO1 is activated in patients with respiratory tract diseases and its deficiency results in the suppression of the Toll-like receptor 3 (TLR3)-dependent epithelial innate immune function and the increase in pathogen uptake [39]. Moreover, in epithelial cells, miRNA-200c overexpression strongly induces the expression of pro-inflammatory IL-6 [40]. Furthermore, it remains unclear if the increase in miRNA-200c levels is a consequence of the progression of disease or the other way round. LDH is known as a marker for cell death, which is enhanced in infectious diseases and routinely recorded to assess the clinical condition of the patients [41]. Here, we demonstrated that elevated LDH levels correlated with enhanced miRNA-200c levels and with the clinical outcome of infection. However, further investigation is needed to evaluate if both markers may be used in conjunction with other predictive markers for COVID-19 such as C-reactive protein (CRP), IL-6, procalcitonin (PCT), D-dimer and serum ferritin [42,43,44,45].

The limitations of the study are the total number of patients and the availability of clinical samples that were collected in the frame of routine diagnostics during the corona pandemic.

COVID-19 patients who have severe cases of the disease often have other medical conditions in addition to the primary infection, such as psychiatric illness, cardiovascular disease or hypertension [46,47]. The use of multiple medications to treat these conditions can lead to drug-to-drug interactions (DDIs), which can cause serious health problems for already debilitated patients [46]. Studies have also shown that not all patients respond equally well to the same treatment, and there are factors that can positively or negatively impact the outcome of treatment [46,47,48]. To reduce the risk of DDIs and find the most appropriate treatment for individual patients, it is important to adopt a personalized approach to healthcare, known as predictive, preventive and personalized medicine (PPPM) [46,48]. Our research has shown that the gene-regulating microRNA-200c is correlated with disease severity and, when combined with other established markers, may help to identify the optimal treatment for individual patients more quickly. Overall, the use of miRNA diagnostic tests in the context of PPPM can help to improve health outcomes by enabling healthcare providers to identify individuals at high risk of developing certain conditions and intervene early to prevent or mitigate their impact.

## 5. Conclusions

In conclusion, we demonstrated that there is a correlation between miRNA-200c and the clinical outcome of COVID-19. Furthermore, enhanced miRNA-200c levels were detected with the progression of disease. The underlying mechanism remains unknown and requires further investigation. Our study provides the basis for further investigations with a larger study cohort to evaluate if miRNA-200c represents a reliable predictive marker for the severity of COVID-19. Consistent with the predictive, preventive and personalized medicine (PPPM/3PM) approach, we showed that the gene-regulating miRNA-200c correlates with disease severity. The combination of these new diagnostic markers with already established markers could enable preventive and individualized therapies in the near future.

## Figures and Tables

**Table 1:** Clinical demographic characteristics of all hospitalized patients and the paired sample group at the time of hospitalization (onset). * *p*-values less than 0.05 are significant; ns: not significant. ** BMI: body mass index; WBC: white blood cell count; RBC: red blood cell count; CRP: C-reactive protein; HCT: hematocrit; Hb: hemoglobin.

Parameter	All Samples “Onset” (*n* = 111)	Paired Samples “Onset” (*n* = 35)	*p* *
Median age (IQR) (years)	64 (53–76)	66 (53–77)	ns
Patient sex (male/female)	72/39	27/8	ns
BMI ** (IQR) (kg/m^2^)	27.8 (24.6–33.3)	28.8 (25.0–31.3)	ns
Recovered/deceased	92/19	24/11	ns
Oxygen therapy (yes/no)	99/12	28/7	ns
Immunosuppressed	7	4	ns
Paraclinical profile (IQR)			
WBC ** (/nL)	6.8 (5.0–9.1)	6.9 (4.9–9.9)	ns
RBC ** (/pL)	4.3 (3.8–4.5)	4.1 (3.8–4.5)	ns
CRP ** (mg/dL)	9.4 (3.8–17.8)	12.4 (5.6–21.2)	0.01
HCT ** (L/L)	0.36 (0.33–0.39)	0.36 (0.32–0.39)	ns
Hb ** (g/dL)	12.3 (11.3–13.2)	12.4 (11.1–12.9)	ns

## Data Availability

The data presented in this study are available on request from the corresponding author.
